# Stimulant medications effects in heat-related illness in ADHD patients: a large database study

**DOI:** 10.3389/fpsyt.2024.1509385

**Published:** 2024-12-20

**Authors:** Samrawit Zinabu, Huda Gasmelseed, Noah Wheaton, Fikirte Girma, Christian Wong, Sair Ahmad Tabraiz, Ayesha Mubasher, Aaron Mack, Patrice Lexima, Ozair Qazi, Ahmad Mohammed, Aseem Sood, Miriam Michael

**Affiliations:** ^1^ Department of Internal Medicine, Howard University, Washington, DC, United States; ^2^ Department of Psychiatry, Addis Ababa University, Addis Ababa, Ethiopia; ^3^ Cardiovascular Department, Mayo Clinic, Rochester, MN, United States; ^4^ Department of Internal Medicine, Mayo Hospital Lahore, Lahore, Pakistan; ^5^ Department of Psychiatry, Howard University, Washington, DC, United States; ^6^ Department of Orthopedics, Howard University, Washington, DC, United States; ^7^ Department of Internal Medicine, University of Maryland, Baltimore, MD, United States

**Keywords:** attention deficit hyperactivity disorder, heat-related illnesses, heat stroke, global warming, central nervous system stimulants

## Abstract

**Introduction:**

Attention Deficit Hyperactivity Disorder (ADHD), a prevalent neurodevelopmental disorder affecting a significant portion of the population, is commonly managed with stimulant medications. These medications, while effective, have been associated with thermoregulatory dysfunction and an increased risk of heat-related adverse events. The current study sought to compare the incidence of such events in ADHD patients receiving stimulant medications with those not on these treatments.

**Methods:**

A retrospective cohort study was conducted utilizing de-identified electronic medical records from a Global Research Network. The study population comprised ADHD patients on stimulant medication aged 6-24 years, with a comparison group of ADHD patients not receiving stimulant medications. Patients were followed from the date of first cohort inclusion (index event) for one year to track heat-related illnesses, including dehydration, hyperthermia, heat stroke, and other heat-related conditions. Propensity score matching was employed to balance baseline characteristics (age, gender) between cohorts. Risk ratios, odds ratios, and hazard ratios were calculated to assess the incidence of heat-related illnesses between groups. Statistical analysis was performed on the TriNetX platform, with survival analysis conducted via Kaplan-Meier estimates.

**Results:**

Analysis revealed a decreased risk of heat-related illnesses in the stimulant medication group, with a risk ratio of 0.559(95% CI: 0.485, 0.644). The mean number of events was also lower in the stimulants medication group (p=0.028). Additionally, Kaplan-Meier survival analysis indicated a higher probability of remaining free from heat-related illnesses in the stimulant group over a one-year period, with a statistically significant difference (log-rank test, χ² = 93.035, p < 0.0001).

**Discussion:**

These results suggest that stimulant medications may be associated with a reduced risk of heat-related illnesses in ADHD patients, potentially contributing to better overall outcomes in this population. Further research is warranted to explore the underlying mechanisms and to confirm these findings across larger and more varied patient populations.

## 1 Introduction

Attention Deficit Hyperactivity Disorder (ADHD), is a prevalent neurodevelopmental disorder affecting a significant portion of the population (1). It is characterized by persistent patterns of inattention, hyperactivity, and impulsivity, with varying presentations across the lifespan (1). Systematic reviews and meta-analyses show that the prevalence of ADHD ranges from 1.6% to 7.6% (2–5). This wide variation in reported prevalence could be due to differences in the methods used across the various studies. ADHD is typically managed with stimulant medications, while effective, have been associated with thermoregulatory dysfunction and an increased risk of heat-related adverse events.

Stimulant medications are generally considered the first-line pharmacological treatment for ADHD; however, there have been observations in some regions indicating a decrease in the use of stimulant medications, accompanied by an increase in the use of non-stimulant medications (6, 7). Stimulants function by modulating dopamine and norepinephrine levels (8). While generally effective, stimulants can induce physiological changes that elevate the risk of heat-related complications (9, 10). These include disruptions in thermoregulation, increased metabolic rate, and alterations in sweating patterns (11).

The mechanisms through which stimulant medications exacerbate heat-related risks are multifaceted. By affecting neurotransmitter levels, they can directly or indirectly impact thermoregulation in warm environments and modify the perception of fatigue and exertion. Stimulants increase sweating but can impair the body’s ability to dissipate heat through alterations in thermoregulatory responses (12). These effects, combined with increased metabolic heat production, heighten the risk of dehydration, hyperthermia, and other heat-related complications, particularly during physical exertion or exposure to high temperatures.

The escalating global temperatures associated with climate change exacerbate the risk of heat-related illnesses (13). Athletes, particularly those taking stimulant medications, are especially vulnerable due to the combined effects of physical exertion, environmental heat, and medication-induced thermoregulatory dysfunction.

While the association between stimulant medications and heat-related adverse events has been documented, there is a paucity of data specifically addressing the incidence of these events in ADHD patients and their clinical significance. Furthermore, the potential for dehydration as a heat-related side effect in this population remains under-explored. To address this knowledge gap, we conducted a descriptive cohort study to assess the risk of heat-related injuries in ADHD patients on stimulant medications and compared the risk with ADHD patients not on stimulant medications, intending to inform preventative measures and clinical practice.

## 2 Methods

### 2.1 Study design

This study employed a retrospective cohort study. This investigated the incidence of heat-related illnesses in ADHD patients prescribed stimulant medications compared to those not receiving such treatment.

#### 2.1.1 Data source

De-identified electronic health records (EHRs) from the TriNetX database, encompassing 20 years of data, were analyzed. TriNetX is a global federated health research network that provides access to electronic medical records, including diagnoses, procedures, medications, laboratory values, and genomic information, from 97 healthcare organizations (HCOs) across 22 countries. These HCOs, which include hospitals, clinics, and academic medical centers, contribute de-identified patient data to the TriNetX platform. The network facilitates research using Natural Language Processing (NLP) to analyze the data. Online HCOs may only provide data in response to specific queries or counts.

The analysis process consisted of two main steps:

1. Defining the Cohorts: Patients were categorized based on specific query criteria into two cohorts:
  - Exposed Cohort (Cohort 1): ADHD patients between the ages of 6 and 24 years with documented prescriptions for stimulant medications (dextroamphetamine, methamphetamine, lisdexamfetamine, amphetamine, serdexmethylphenidate, dexmethylphenidate and methylphenidate) between January 1, 2022, and December 31, 2022.
  - Comparison Cohort (Cohort 2): ADHD patients between the age of 6 and 24 years who are not on stimulant treatment (dextroamphetamine, methamphetamine, lisdexamfetamine, amphetamine, serdexmethylphenidate, dexmethylphenidate and methylphenidate). This group could be receiving non stimulant medications or not receiving any pharmacological treatment.
2. Setting up and Running the Analysis: This involved defining the index event, the time frame, and outcome criteria.
  - Index Event: The point in time when each patient enters the analysis, defined as the first occurrence of the selected cohort criteria.
  - Time Window: Outcomes were analyzed from the same day as the index event and continued for one year.
  - Outcome: The primary outcome of interest was the occurrence of any heat-related illnesses within one year after being included in the study population. These illnesses were identified using the following ICD codes:
    - Heat syncope (R55)
    - Heat cramps (G73.0)
    - Heat exhaustion (T67.2)
    - Heat fatigue (R53.0)
    - Heat edema (T67.4)
    - Hyperthermia (T28.0 - General hyperthermia; T67.0 - Heat-related hyperthermia)
    - Dehydration (E86.0)
    - Rhabdomyolysis (M62.82)
    - Heat stroke, sunstroke, and exertional heat stroke (T67.0)
    - Other effects of heat and light (T67.9 - Unspecified heat-related effect)

### 2.2 Cohort construction

The initial cohort consisted of 1,082,112 participants enrolled between January 1, 2022, and December 31, 2022. After applying inclusion and exclusion criteria, the final study population was divided into two groups: the exposed cohort (Cohort 1) consisted of ADHD patients with documented prescriptions for stimulant medications (amphetamine, dextroamphetamine, methamphetamine, lisdexamfetamine, serdexamethylphenidate, dexmethylphenidate or methylphenidate). This query was run on the network Research with 93 health care organizations (HCOs) queried, and a total of 64 providers responded with patients. The comparison cohort (Cohort 2) included ADHD patients without the above-listed stimulant medication prescriptions. This query was run on the network Research with 93 HCO(s) queried, and a total of 83 providers responded with patients.

Inclusion and exclusion criteria

- Inclusion Criteria: The study encompassed individuals of both genders aged 6 to 24 years and diagnosed with ADHD based on the specified ICD-10 codes.
- Exclusion Criteria: The study excluded individuals under the age of 6 years and above 24 years. Those with known risk factors for heat-related illnesses were also excluded. These included individuals who are taking one or more of the medications such as antipsychotics, anticholinergics, antihistamines, antidepressants, carbonic anhydrase inhibitors, other CNS stimulants, antihypertensives, diuretics, beta-blockers, calcium channel blockers, angiotensin-converting enzyme inhibitors and angiotensin receptor blockers and/or who are having the conditions like cocaine/alcohol dependence, alcohol abuse, Sjogren syndrome, thyrotoxicosis, pheochromocytoma, anhidrosis, and pregnancy (14, 15).

### 2.3 Data analysis

Statistical analysis was performed using the TrinetX platform. Propensity score matching was employed to balance baseline characteristics between the two cohorts, ensuring comparability in the absence of random assignment to treatment or intervention. The matched characteristics include age at index and gender. Measures of association analysis were utilized to see the risk of heat-related illnesses between the groups. The number of outcome events that occurred within the time window in each group was described, and the mean number of outcome events was calculated for those with outcomes during the study period using a number of instances analysis. The number of instances was grouped by visit, which counts any visit for the outcome as one, regardless of how many times it occurred. Additionally, Kaplan-Meier estimates were used to assess survival probability at the end of the study period, and hazard ratios were calculated using the same analysis.

### 2.4 Ethical considerations

This retrospective study is exempt from informed consent. The data reviewed is a secondary analysis of existing data, does not involve intervention or interaction with human subjects, and is de-identified according to the de-identification standard defined in the HIPAA Privacy Rule.

## 3 Results

**Figure 1:**
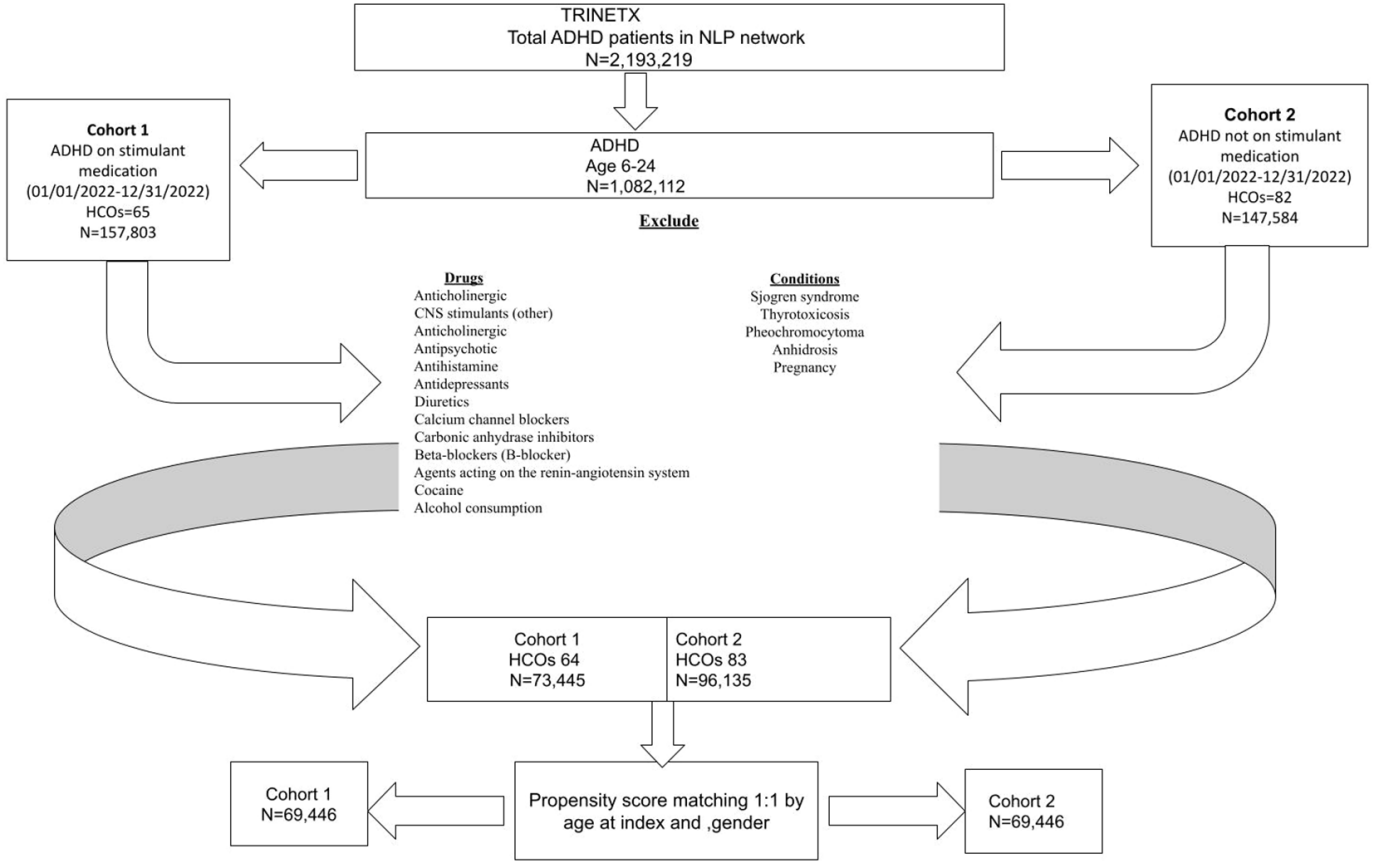
A flowchart of the cohort construction.

**Figure 2:**
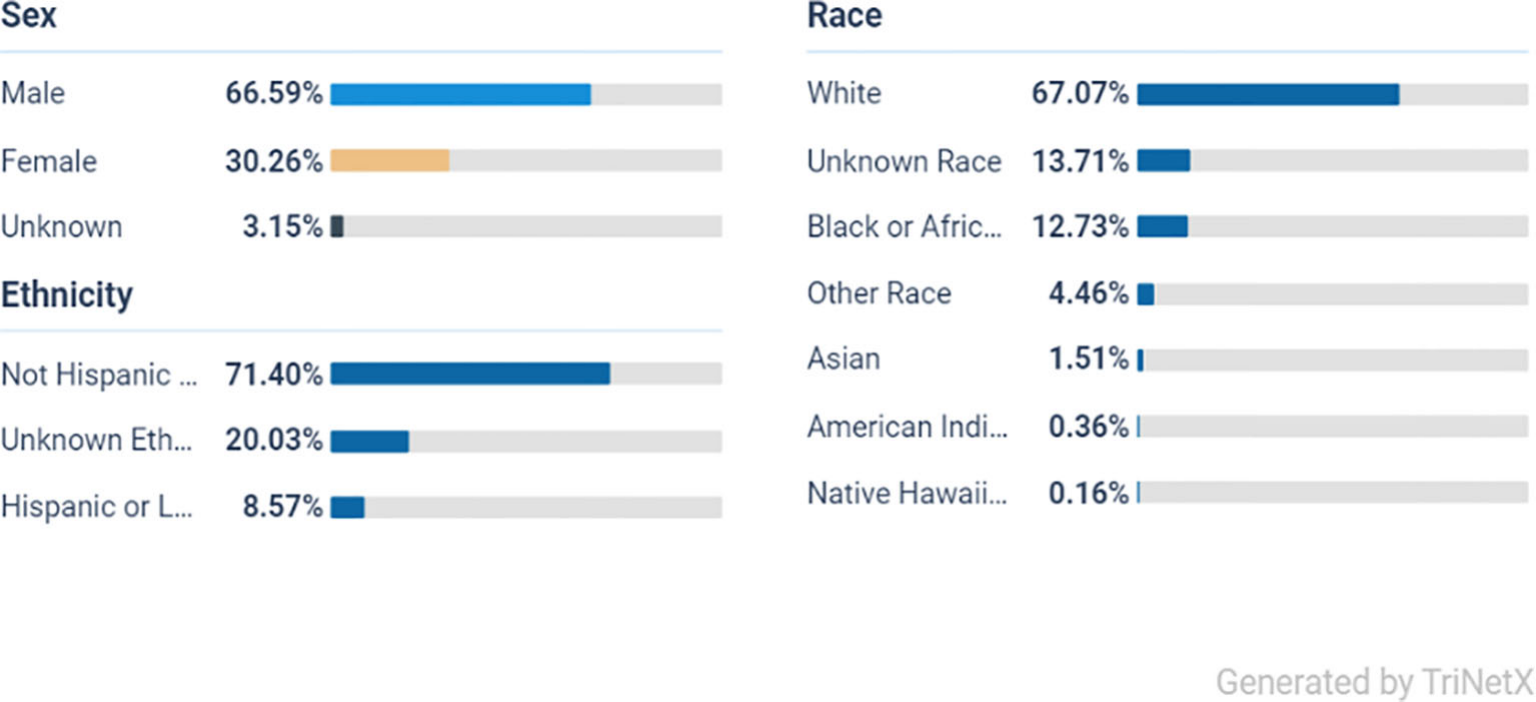
Demography of ADHD on stimulant medication (cohort 1).

**Figure 3:**
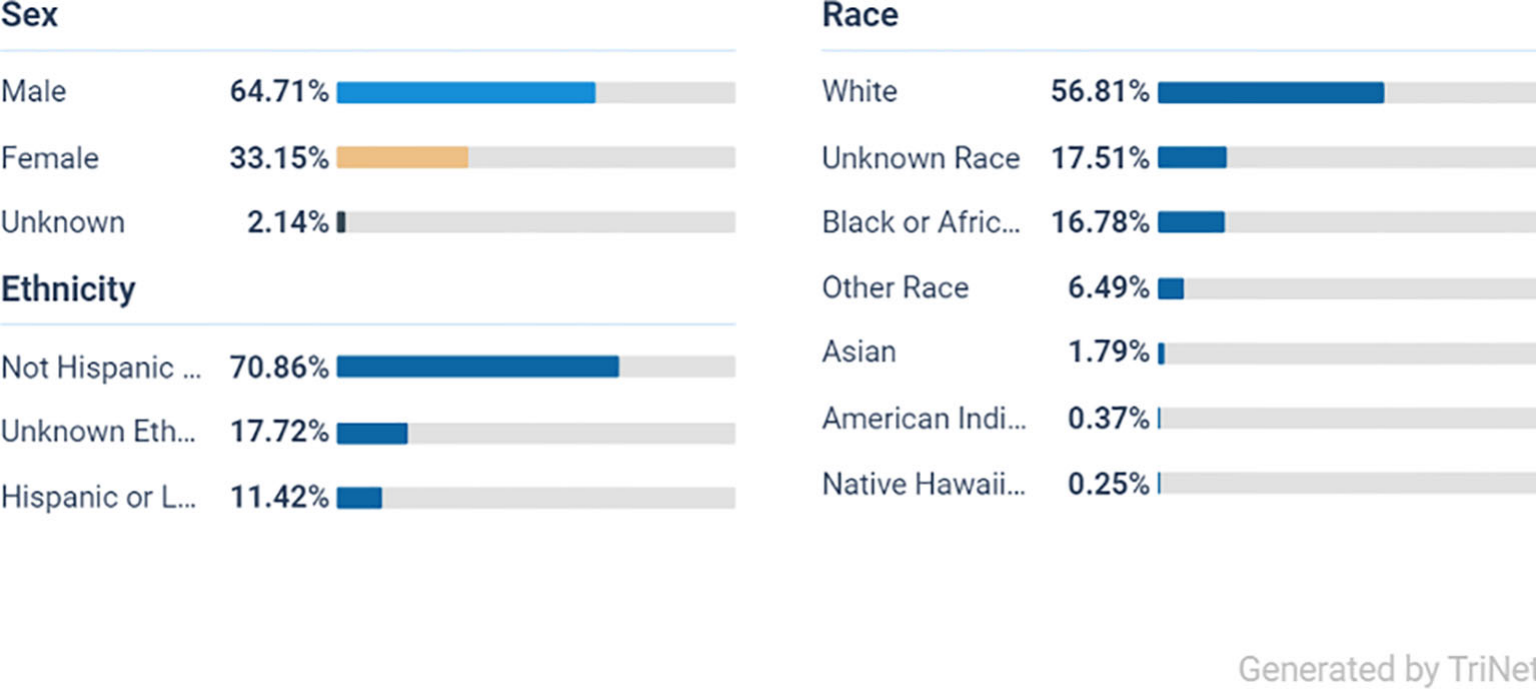
Demography of ADHD, not stimulant medication (cohort 2).

From the initial population of 2,193,219 ADHD patients, 1,082,112 met the age criteria (**Figure 1**). After applying the inclusion and exclusion criteria, Cohort 1 included 73,445 patients who received a prescription for one or more of the listed stimulant medications, while Cohort 2 included 96,135 patients who did not receive any of these medications (**Figure 1**). Ethnically, the majority of the ADHD population in both cohorts are White persons, followed by Black or African American persons, other races, and Asian groups, indicating a broad presence across diverse populations (**Figures 2**, **3**).

From a total of 73,445 participants in the stimulant group, the majority were male at 66% (48,470) and White person at 67% (49,208), Black or African American persons at 12.7% (8,813), and the smallest group were Native Hawaiian persons at 0.16% (117).

From a total of 96,135 participants in the non-stimulant group the majority were male at 66% (63,452) White persons at 56% (53,766), Black or African American persons at 16% (15,420), and the smallest group were Native Hawaiian persons at 0.25% (240).

Following 1:1 propensity score matching by age at index and gender to ensure comparability of baseline characteristics, the cohorts were balanced, with each cohort comprising 69,446 patients (**Table 1**). The matched cohorts exhibited evenly distributed differences in age at index and gender.

**Table 1:** Characteristics after propensity score matching.

Demographics	Taking stimulants	Not taking stimulants	Std. Diff	P value
Age at Index, Mean	12.6+/-4.5	12.6+/-4.5	0.002	0.683
Female,%	30.3	30.2	0.002	0.77
Male,%	66.9	66.9	0.002	0.776

There were 299 instances of heat-related illness in cohort 1 and 535 in cohort 2. Subsequent outcome analysis revealed statistically significant differences in the risk of heat-related illnesses between the two cohorts. The risk difference was -0.003 (95% CI: -0.004, -0.003), the risk ratio was 0.559 (95% CI: 0.485, 0.644), and the odds ratio was 0.557 (95% CI: 0.483, 0.642), indicating a reduced risk in patients taking stimulant medications (**Table 2**). Upon analyzing individual heat-related illnesses, we found statistically significant reductions in risk for both dehydration and rhabdomyolysis in patients on stimulant group, with risk ratios of 0.563 (95% CI: 0.486–0.651) and odds ratio of 0.564 (95% CI: 0.335–0.951), respectively (**Table 3**). However, no significant risk reduction was observed for heat syncope, heat exhaustion, heat stroke, sunstroke, or other effects of heat and light (**Table 4**).

**Table 2:** Comparison of risk in ADHD patients with and without stimulant medication.

Risk		Risk Difference	Odds Ratio	Risk Ratio
Stimulant Medication Group	Non- Stimulant Medication Group			
0.004	0.008	-0.003 (-0.004, -0.003)	0.557 (0.483,0.642)	0.559 (0.485,0.644)

**Table 3:** Risk analysis for individual of heat-related illnesses.

After propensity score matching												
Outcome	Cohort	Patients in cohort	Risk	Risk difference with95%CI	P-value	Risk Ratio	P-value of Risk Ratio	Kaplan-Meier	Log-rank Test with P-value of Kaplan	Hazard Ratio with 95 CI	P-value of Hazard Ratio	Mean of number of Instance
Dehydration	ADHD on stimulants	279	0.004	-0.003(-0.004,-0.002)	** **	0.563 (0.486, 0.651)	N/A	99.56%	85.095(0.000)	0.508(0.438,0.588)	0.002	1.29
	ADHD not on stimulants	496	0.007					99.18%				1.597
Rhabdomyolysis	ADHD on stimulants	22	0	-0.000 (-0.000, -0.000)		0.564 (0.335, 0.951)	N/A	99.70%	6.338	0.517 (0.306,0.872	0.388	1.955
	ADHD not on stimulants	39	0.001					99.94%				1.538
Heat Syncope	ADHD on stimulants	10	0	0 (-0.000, 0.000)		1 (0.416, 2.402)	N/A	100%	0.385	0.476	0.096	2
	ADHD not on stimulants	10	0					100%				1
Heat Stroke and sunstroke	ADHD on stimulants	0	0	-0.000 (-0.000, -0.000)	0.002	N/A		100%	1	–	–	–
	ADHD not on stimulants	10	0					100%				1
Heat Exhaustion	ADHD on stimulants	10	0	-0.000 (-0.000, 0.000)	1	1 (0.416,2.402)	N/A	99.99%	3.426	0.351 (0.110,1.119)	0.741	–
	ADHD not on stimulants	10	0					99.98%				1
Other effects of heat and light	ADHD on stimulants	10	0	0 (-0.000, 0.000)	1	1 (0.416,2.402)	N/A	100%	0.011	0.864 (0.054,13.807)		–
	ADHD not on stimulants	10	0					100%				1

**Table 4:** Comparison of mean difference.

Number of Instances					
Cohort	Patients in cohort	Patients with outcome	Mean	Standard Deviation	Median
ADHD on Stimulant medication	299	69446	299	1.341	1
ADHD not on stimulant medication	535	69,446	535	1.538	1
	t	df		p	
Test Statistics	-2.196	832		0.028	

Kaplan-Meier survival analysis (**Table 5**) indicated that 99.52% of patients presumed to be taking stimulant medications had no outcome of interest within a 1-year study period following the index event compared to 99.09% of those not taking stimulant medications. The log-rank test showed a significant difference (χ2 = 93.035, df = 1, p < 0.0001). The hazard ratio for heat-related illnesses in the stimulant medication cohort was 0.505 (95% CI: 0.438 to 0.582; p = 0.0047), indicating the risk reduction is statistically significant. In summary, the hazard and risk ratios suggested a decreased risk of heat-related illnesses in the stimulant medication group. The Kaplan-Meier survival analysis indicates that ADHD patients prescribed stimulant medication have a significantly better survival probability, which means they have better survival free from heat-related illnesses compared to those not on stimulant medication.

**Table 5:** Kaplan-Meier survival analysis.

		cohort statistics			
	cohort	Patients in Cohort	Patients with Outcome	Median Survival (Days)	Survival Probability at End of Time Window
1	ADHD on Stimulant medication	69446	299		99.52%
2	ADHD not on Stimulant medication	69446	535		99.087
Log- Rank Test					
X^2^		df		p	
93.035		1		<0.0001	

The number of instances analysis for the average count of heat-related illnesses per participant showed patients in the stimulant medication group experienced a slightly lower average number of heat-related illnesses (1.341) compared to those in the non-stimulant group (1.538) (**Table 4**). The standard deviation values indicate a similar level of variability in the number of heat-related illnesses in both groups. Despite the difference in mean values, both groups had the same median number of heat-related illnesses, which was one. This suggests that while the central tendency of illness distribution is identical, the stimulant group had fewer extreme values, resulting in a lower mean. This explains the statistical finding that the stimulant medication group experienced fewer heat-related illnesses on average compared to the non-stimulant group, with the difference being statistically significant.

A t-test was performed to compare the means, yielding a t-statistic of -2.196 with 832 degrees of freedom and a p-value of 0.028. This indicates that the difference in the mean number of heat-related illnesses between the two groups is statistically significant (**Table 4**).

## 4 Discussion

This study explored the relationship between stimulant medications, commonly prescribed for ADHD, and the risk of heat-related illnesses. By comparing ADHD patients who are prescribed stimulant medications with those not receiving such treatment, we aimed to understand how these medications impact thermoregulation and the incidence of heat-related complications. Stimulant medications, such as amphetamines and methylphenidate, have been shown in prior studies to increase metabolic rates, potentially raising the risk of conditions like hyperthermia, dehydration, heat stroke, heat syncope, heat exhaustion, heat cramps, and rhabdomyolysis (16). However, our findings suggest a different outcome in clinical settings.

Our results revealed a reduced risk of heat-related illnesses in ADHD patients on stimulant medications compared to those not taking these medications. This contrasts with previous studies and animal models that reported an increased risk (17–19). The risk ratio also indicates a 44% lower risk of heat-related illnesses in the stimulant group. This unexpected outcome may reflect differences between clinical and experimental environments, suggesting that in real-world settings, stimulant medications may have a protective effect.

The Kaplan-Meier survival analysis supported these results, showing that patients on stimulant medications had a slightly higher probability of remaining free from heat-related illnesses compared to the non-stimulant group. Several factors could explain these results. Stimulant medications are known to increase sweating and thirst, which may help counteract the elevated metabolic rate and core body temperature, reducing the risk of dehydration and rhabdomyolysis. Animal studies have shown similar patterns, where rats treated with amphetamines overcompensated for fluid loss by drinking more, even without thirst signals (20).

Another explanation could be that ADHD patients on stimulants tend to have better symptom control, leading to less impulsive behavior, which may reduce their exposure to excessive heat or situations that could precipitate heat-related illnesses (21, 22). Moreover, ADHD patients are often advised to stay hydrated and avoid diuretics such as coffee, energy drinks, and tea, especially when on stimulant medications, which could explain the lower incidence of heat-related adverse effects in this group (20).

Our findings also suggest that stimulant medications could have an impact on body mass index (BMI), which plays a role in heat-related risks. ADHD patients tend to have a higher BMI than the general population, increasing their vulnerability to heat-related illnesses (23, 24). However, stimulant medications are associated with a reduction in BMI, which may help lower the risk of heat-related injuries (24). This aligns with our analysis of heat-related illness instances, which revealed a significant difference in the average number of events between the two groups. Patients not on stimulants experienced a higher initial risk and more frequent subsequent heat-related illnesses, while the stimulant group had fewer extreme cases, contributing to a lower mean number of illnesses (1.341 *vs*. 1.538).

We also found a lower incidence of dehydration among ADHD patients on stimulant medications compared to those not receiving stimulants. This could be attributed to improved self-regulation and better hydration practices in the stimulant group, effectively reducing the risk of dehydration. Additionally, there was a significant reduction in the risk of rhabdomyolysis in the stimulant group. This contrasts with earlier research suggesting that high doses of amphetamines increase the risk of rhabdomyolysis due to excessive neuromuscular stimulation and depletion of energy stores in muscle cells (25, 26). One possible explanation is that patients in our study may not have reached the high stimulant concentrations associated with increased rhabdomyolysis risk, thereby reducing the likelihood of this serious complication.

The absence of observed protective effects for other heat-related illnesses during individual analysis raises important questions. Given that hydration is considered a key factor in preventing many heat-related illnesses, including heat exhaustion and heat stroke, it would seem logical that preventing dehydration might also confer protection against these more severe illnesses. However, the lack of a significant association could be explained by several factors. First, heat-related illnesses such as heat exhaustion and heat stroke involve not just hydration status but also thermoregulation and environmental factors such as ambient temperature and humidity. While dehydration exacerbates the risk, it is not the sole determinant of these conditions. In contrast, rhabdomyolysis may be more directly linked to dehydration and the resultant electrolyte imbalances, explaining the observed protective effect.

Another possible explanation is that stimulant use might not sufficiently influence thermoregulatory mechanisms in the way it impacts hydration. Stimulants primarily affect neurochemical pathways related to attention, arousal, and behavior, which might improve hydration practices but not directly impact the body’s ability to regulate temperature in extreme heat.

These findings have important clinical implications. The reduced risk and incidence of heat-related illnesses in ADHD patients on stimulant medications suggest that these treatments may mitigate some of the risks associated with thermoregulation in this population. Potential mechanisms for this protective effect include lower BMI, increased fluid intake, and improved attention and behavior, which may lead to better self-care and adherence to preventive measures against heat-related illnesses. This is particularly relevant given that previous reports have linked stimulant medications to an increased susceptibility to heat-related injuries and hyperthermia (21).

In summary, our study challenges some of the assumptions from earlier literature by showing a protective effect of stimulant medications in preventing heat-related illnesses. These results underscore the need for careful clinical management of ADHD patients, particularly in environments with high heat exposure, and highlight the importance of hydration and other preventive measures in this population.

### 4.1 Limitations and future directions

This study has several limitations that must be considered when interpreting the results. First, the retrospective design may introduce biases, particularly regarding data collection and patient selection. For example, we relied on electronic health records, which only captured patients who sought medical attention for heat-related illnesses. As a result, milder cases of heat-related conditions that did not require medical intervention may have been underreported, potentially skewing the data. Additionally, we excluded patients with pre-existing conditions, which may limit the generalizability of our findings to broader ADHD populations, particularly those with comorbidities.

We also could not account for several confounding factors that may influence the risk of heat-related illnesses, including environmental conditions (e.g., temperature and humidity), physical activity levels, hydration status, and other medications. These variables are crucial, as they can interact with stimulant medications and influence thermoregulatory function. Furthermore, the study did not control for patients who may have switched between stimulant and non-stimulant treatment groups during the one-year study period, which could affect the outcomes.

In this study, efforts were made to mitigate the impact of polypharmacy by excluding patients on medications known to affect thermoregulation or increase the risk of heat-related illnesses. However, this exclusion criterion may not account for all other medications that could indirectly influence thermoregulatory function.

The study lacks detailed clinical information regarding how ADHD diagnoses were made, the severity of ADHD within each group, and whether patients actively adhered to stimulant therapy. The designation of patients as being “on stimulants” is based solely on prescription data, with no confirmation of medication adherence or usage at the time of heat-related events.

Furthermore, there may be unmeasured confounding factors, such as differing baseline severities of ADHD between treated and untreated groups, which could influence the outcomes. The absence of a control group of ADHD patients on non-stimulant treatments also limits our ability to delineate whether the perceived benefit is specific to stimulant use or to receiving any form of treatment for ADHD.

Additionally, the database does not provide context for dehydration diagnoses, leaving ambiguity as to whether these cases were specifically heat-related or related to other conditions (e.g., viral illnesses).

Another limitation is the potential for selection bias in the matching process. Although the 1:1 propensity score matching by age and gender helped ensure balanced cohorts, there may still be unmeasured variables that could have impacted the results. Future research should address these limitations by conducting prospective studies that track environmental exposure, hydration practices, physical activity, and medication use in real-time. Additionally, investigating the biological mechanisms by which stimulant medications may reduce the risk of heat-related illnesses could provide more insights into their protective effects. Expanding the study to include more diverse populations, including those with pre-existing conditions, would also enhance the generalizability of the findings.

## 5 Conclusion

In conclusion, this study suggests that stimulant medications may not increase the risk of heat-related illnesses in ADHD patients and may contribute to a lower incidence of such conditions compared to non-stimulant users. These findings are particularly relevant given the growing concerns about rising global temperatures and the potential vulnerability of ADHD patients to heat-related risks. However, these results must be interpreted with caution. The study’s retrospective nature and reliance on electronic medical records introduce limitations, including the potential for unmeasured confounding factors. Ultimately, while this study offers valuable insights into the relationship between stimulant use and heat-related illnesses in ADHD patients, additional research is essential to fully understand the implications of these findings, particularly in the context of climate change and increasing global temperatures.

## Data Availability

The original contributions presented in the study are included in the article/supplementary material. Further inquiries can be directed to the corresponding author.
